# Risk Assessment of kINPen Plasma Treatment of Four Human Pancreatic Cancer Cell Lines with Respect to Metastasis

**DOI:** 10.3390/cancers11091237

**Published:** 2019-08-23

**Authors:** Sander Bekeschus, Eric Freund, Chiara Spadola, Angela Privat-Maldonado, Christine Hackbarth, Annemie Bogaerts, Anke Schmidt, Kristian Wende, Klaus-Dieter Weltmann, Thomas von Woedtke, Claus-Dieter Heidecke, Lars-Ivo Partecke, André Käding

**Affiliations:** 1 ZIK plasmatis, Leibniz Institute for Plasma Science and Technology (INP Greifswald), Felix-Hausdorff-Str. 2, 17489 Greifswald, Germany; 2 National Centre for Plasma Medicine (NZPM), Langenbeck-Virchow-Haus, Luisenstr. 58/59, 10117 Berlin, Germany; 3 Department of General, Visceral, Thoracic, and Vascular Surgery, Greifswald University Medical Center, Ferdinand-Sauerbruch-Str., 17475 Greifswald, Germany; 4 PLASMANT, Chemistry Department, University of Antwerp, 2610 Antwerp, Belgium; 5 Solid Tumor Immunology Group, Center for Oncological Research, University of Antwerp, 2610 Antwerp, Belgium; 6 Institute for Hygiene and Environmental Medicine, Greifswald University Medical Center, Walther-Rathenau-Str. 48, 17489 Greifswald, Germany

**Keywords:** cell adhesion, plasma medicine, oncology

## Abstract

Cold physical plasma has limited tumor growth in many preclinical models and is, therefore, suggested as a putative therapeutic option against cancer. Yet, studies investigating the cells’ metastatic behavior following plasma treatment are scarce, although being of prime importance to evaluate the safety of this technology. Therefore, we investigated four human pancreatic cancer cell lines for their metastatic behavior in vitro and in chicken embryos (in ovo). Pancreatic cancer was chosen as it is particularly metastatic to the peritoneum and systemically, which is most predictive for outcome. In vitro, treatment with the kINPen plasma jet reduced pancreatic cancer cell activity and viability, along with unchanged or decreased motility. Additionally, the expression of adhesion markers relevant for metastasis was down-regulated, except for increased CD49d. Analysis of 3D tumor spheroid outgrowth showed a lack of plasma-spurred metastatic behavior. Finally, analysis of tumor tissue grown on chicken embryos validated the absence of an increase of metabolically active cells physically or chemically detached with plasma treatment. We conclude that plasma treatment is a safe and promising therapeutic option and that it does not promote metastatic behavior in pancreatic cancer cells in vitro and in ovo.

## 1. Introduction

Ninety percent of cancer deaths are not caused by the primary tumor but because of metastasis [1]. For this to happen, individual cancer cells leave the primary tumor (or later: metastatic sites) into the bloodstream or lymphatic vessels to settle at distance organs or lymph nodes, respectively [2]. Therapies, therefore, should not only be effective in diminishing tumor growth but also avoid spurring of metastasis due to treatment. In the last decade, traditional therapies such as surgery, chemotherapy, and radiotherapy have been increasingly complemented with novel therapeutic approaches. This includes systemic treatments, for example, immunotherapies, small molecules, and other biological [3,4,5] as well as local treatments such as electrochemotherapy, photodynamic therapy, and a novel concept involving the application of cold physical plasma [6,7,8,9,10].

Cold physical plasma is a partially ionized gas that expels a variety of reactive oxygen and nitrogen species into the ambient air [11]. As these sources can be operated at body temperature, they can be applied to human tissue without causing thermal damage. Recent in vitro [12,13,14] and in vivo [15,16,17] evidence suggests a tumor-toxic potential of cold physical plasma sources, with reactive species playing a significant role in the effects observed. First, studies reported on beneficial antitumor effects of cold physical plasma in the palliation of cancer patients within clinical observational case studies [18,19,20,21]. These results were achieved using an atmospheric pressure argon plasma jet (kINPen MED) accredited as a medical device in Europe [22].

While such a novel approach generates excitement among researchers and practitioners, new technologies and therapies should be effective and safe. The kINPen plasma jet has been investigated for genotoxic safety, and several studies—partially based on genotoxicity testing according to OECD-based protocols—have shown that there is no evidence of mutagenic effects on human cells in vitro and in HET-CAM tests using chicken embryos (in ovo) [23,24,25]. The source can also be applied without thermal harm and is well-tolerated in patients without any severe adverse events noted [26,27,28,29]. At the same time, we reported a lack of tumor formation in a 1-year follow-up study in mice that had received six repetitive plasma-treatment sessions [30]. However, a point that has so far not been addressed is whether cold physical plasma treatment may promote metastasis due to physically or chemically dislodging cells from their primary tumor or by changing the cell’s adhesion molecule profile.

Using the kINPen plasma jet and four human pancreatic cancer cell lines, we investigated potentially metastasis-promoting effects in vitro in two-dimensional cultures, three-dimensional cultures, and three-dimensional cultures grown on chicken embryos, a realistic model allowing tumor formation with vascularization. Despite each of the cell lines showing an individual behavior in response to plasma treatment as seen with several biological assays, we could not identify a metastasis-promoting effect when exposing four human pancreatic cancer cell lines to the plasma of the kINPen argon jet.

## 2. Materials and Methods

### 2.1. Cell Culture

Pancreatic cancer cell lines (MiaPaCa2, PaTuS, PaTuT, and Panc01) were cultured in *Dulbecco’s modified Eagle’s Medium* (DMEM, Pan Biotech, Aidenbach, Germany) supplemented with 10% fetal bovine serum (FCS), 2% glutamine, and 1% penicillin/streptomycin (all Sigma, Steinheim, Germany). For incubation, cells were placed at 37 °C and 5% CO_2_ in a humidified cell culture incubator (Binder, Tuttlingen, Germany). For in-vitro experiments with 2D cell cultures, 2 × 10^4^ cells were seeded in 100 µL of *Roswell Park Memorial Medium* (RPMI, Pan Biotech) also supplemented with FCS, glutamine, and penicillin-streptomycin, in tissue culture-treated 96-well flat-bottom plates (Eppendorf, Hamburg, Germany). Cell counting was performed in a highly standardized fashion by determining the absolute number of cells using the *Attune NxT* flow cytometer (Thermo Scientific, Waltham, MA, USA) and propidium iodide (PI; Sigma) for live-dead discrimination. For optimal culture conditions, the rim of the Eppendorf plates was filled with double-distilled water to prevent excessive evaporation of culture medium in the outer wells (edge effect).

### 2.2. Cold Physical Plasma and Treatment Regimen 

For treatment with cold physical plasma, a *kINPen* atmospheric pressure plasma jet (Neoplas, Greifswald, Germany) was utilized at room temperature. The device was operated with 99.999% pure argon gas (Air Liquide, Paris, France) at 2 standard liters per minute (SLM). Mock treatment with argon gas alone (plasma off) was carried out to control for any potential effect of argon gas on cells alone (argon controls), while untreated controls were exposed neither to plasma nor to argon gas. In-vitro treatment of 2D cell cultures in flat-bottom plates or of spheroids (see below) in ultra-low-affinity (ULA) plates (PerkinElmer, Waltham, MA, USA) were carried out utilizing a computer-controlled xyz-table (CNC-Step, Geldern, Germany). This table works with specific software (WinPC-NC) that standardizes the distance of the plasma effluent to the cells (12 mm = distance nozzle to cells), velocity, as well as the treatment time that was set to 60 s for treatment with plasma or argon gas. During in-vitro treatment, cells were cultivated in RPMI culture medium that remained on the cells afterward. Evaporation through the jet effluent was measured via precision balance (Sartorius, Göttlingen, Germany) and was resubstituted with 12 µL of double-distilled water per treated well. Tumors growing on the chorion-allantois membrane of eggs (TUM-CAM, see below) were treated manually for 60 s plasma at 9 mm distance nozzle-to-target (the tip of the plasma effluent touching tumor surface). Detached cells (floaters) were collected post-treatment immediately, and a separate treatment of them was not performed. 

### 2.3. Quantification of Metabolic Activity

In-vitro treated cells growing in 2D cultures were incubated for 24 h after their initial exposure to the plasma effluent or argon gas before the addition of 7-hydroxy-3H-phenoxazin-3-on-10-oxid (resazurin, Alfa Aesar, Haverhill, MA, USA) that is transformed by viable cells to the fluorescent resorufin. Fluorescence was measured 4 h after incubation with the dye utilizing a multiplate reader (Tecan F200, Männedorf, Switzerland) at λ_ex_ = 530 nm and λ_em_ = 590 nm to quantify the number of metabolically active cells. To validate the importance of plasma-derived reactive oxygen species (ROS), the antioxidant n-acetylcysteine (NAC, final concentration 2 mM; Sigma) was added to control experiments. To harvest cells that have detached either naturally or potentially through plasma treatment (floaters), the cell culture supernatant was collected immediately after treatment and added to a new plate. This new plate was incubated for 6 further days under optimal growing conditions before resazurin was added to quantify the amount of metabolically active cells in these wells. A similar protocol was used to identify the number and metabolic activity of floaters collected during in-ovo experiments.

### 2.4. Culture and Analysis of 3D Tumor Spheroids

Before utilizing each of the four human pancreatic cancer cell lines for tumor spheroid formation, they were stained with the cell tracing reagent 1,1′-Dioctadecyl-3,3,3′,3′-tetramethylindocarbocyanine perchlorate (DiL; Thermo Fisher, Waltham, USA). Afterward, 3 × 10^3^ cells were seeded in ULA 96-well plates in RPMI containing 0.24% methylcellulose (Methocel; Sigma Aldrich, Steinheim, Germany). To form spheroids, they were centrifuged for 10 min at 1000× *g*. After 3 days of incubation in the incubator, the fresh culture medium was added, and the plasma treatment was performed as described above. After 4 h of incubation, the medium was removed, leaving only 50 µL per well. To this medium, 50 µL of Matrigel (LDEV-free; concentration 2 mg/mL; Corning, New York, NY, USA) extracellular matrix component was added. The plate was centrifuged and incubated for one further hour before 100 µL fresh medium containing sytox green (Thermo Fisher) dead cell staining solution was added on top of the Matrigel. 

### 2.5. High Content Imaging

All images were acquired using a *high content imaging* device (Operetta CLS; PerkinElmer) that utilizes a 16-bit sCMOS camera with 4.7 megapixels, laser-based autofocus, high-speed precision *xyz*-table, and eight different excitation light sources and emission wavelengths via bandpass filters. Time-lapse imaging experiments were carried out under environmental conditions set to 37 °C and 5% CO_2_ during a 4-h time course. The water-filed rim of the 96-well plates protected the cells from excessive evaporation. Bright-field and digital phase contrast (DPC) images were acquired with a 20 × air objective (NA = 0.4; Zeiss, Jena, Germany) for 25 fields of view (FOV) per well with six technical replicate wells. Imaging frequency was every 20 min, which resulted in a quantitative analysis of around 3 × 10^4^ single cells per cell line, per condition, and per time point. Spheroids were imaged immediately and at 24 h, 48 h, and 72 h post-plasma treatment with a 5 × air objective (NA = 0.16; Zeiss, Jena, Germany) in confocal mode with seven z-stacks per spheroid. Brightfield and fluorescence channels (λ_ex_ = 530–560 nm and λ_em_ = 570–650 nm for detection of the pancreatic cancer cells labeled with DiL; λ_ex_ = 460–490 nm and λ_em_ = 500–550 nm for dead cell quantification of cells positive for sytox green; and λ_ex_ = 435–460 nm and λ_em_ = 470–515 nm to detect the spheroids’ autofluorescence) were acquired. The experimental setup and image quantification were performed with the image acquisition and analysis software Harmony 4.8 (Perkin Elmer, Waltham, MA, USA). For analysis of time-lapse experiments, at least 1500 individual cells per condition and cell line were tracked with their pseudo-cytosolic signal (DPC channel) to assess the individual cell’s motility based on algorithm detection methods. In parallel, cell counts as well as cell growth area (cell cluster area in PaTuS cells) were calculated throughout the time course. For spheroid analysis, the brightfield channel was inverted and merged with fluorescence channels. This calculated image was further processed, and an intensity cut-off was applied to detect the spheroid area independent of surrounding cells. Quantification of the metastatic cancer cells outside the spheroid region was performed via segmentation of their DiL signal, whereas presumable objects with higher sum fluorescence intensities of 2 × 10^5^ were excluded from analysis (= autofluorescent conglomerates). For calculation of viability, objects with mean fluorescence intensity of sytox green higher than 3 × 10^3^ were considered to be dead cells. For investigating the spheroids symmetry, a polynomial function was utilized ($R_{nm}\;\left(\rho ,\varphi \right)=\;\rho^{n}\;e^{-im\varphi }$). Threshold compactness was investigated by calculating the relation of objects inside the spheroid (with intensities of 60% of the maximum) concerning the spheroid border using the formula: $c=\frac{\sqrt[{2}]{s\;body}}{s\;border}$. The spheroid’s profile is defined by the shortest distance from the border to higher cell intensities.

### 2.6. Flow Cytometry

Different molecules at the cell membrane important for cell adhesion were quantified via flow cytometry. Cells were harvested 4 h and 24 h post-plasma exposure with the enzyme accutase (BioLegend, San Diego, CA, USA) and stained with monoclonal antibodies targeting the anti-cluster of differentiation (CD) 49b conjugated with phycoerythrin (PE), anti-CD49d PE-Cy7, anti CD324 (E-cadherin) Alexa Fluor (AF) 488, and anti-CD326 (EpCam) Brilliant Violet (BV) 605 as well as 4,6-Diamidin-2-phenylindol (DAPI) (all BioLegend). After washing with phosphate-buffered saline (PBS, PAN Biotech), cells were acquired with a Cytoflex S (Beckman-Coulter, Brea, CA, USA) flow cytometer. Following doublet discrimination, cells were gated via their size and granularity with forward and side scatter (FSC, SSC). Only DAPI^-^ (viable) cells were used for quantification of mean fluorescence intensities of cell surface integrins (CD49b, CD49d) and cadherins (CD324, CD326) on the four human pancreatic cancer cell lines. Unstained and treated as well as unstained and untreated controls helped to create autofluorescence vectors for accurate quantification of expression values. To enumerate floaters, supernatants were mixed with fixation and permeabilization buffer (BioLegend) containing DAPI to count all nucleated cells sensitively, accurately, and quantitatively using flow cytometry. Data analysis of more than 2000 flow cytometry acquisitions across all assays was performed using Kaluza 2.1.1 software (Beckman-Coulter).

### 2.7. In ovo Experiments

After delivery, fertilized chicken eggs (Valo BioMedia, Osterholz-Scharmbeck, Germany) were incubated at constant temperatures around 37.7 °C and 65% humidity in an egg incubator with automatic turning function. To prepare the chorion-allantois membrane (CAM) for tumor cell implantation 6 days later, the upper pole was disinfected, and the eggshell was punctured with a sterile cannula without damaging the inner components. During all steps outside the incubator, a heat block with a custom-made egg adapter (Stuart Scientific, Staffordshire, UK) was utilized to ensure regular maturation. The lesion was closed with sticking plaster to prevent contamination, and the egg was again placed in the incubator but without frequent turning. On day 8, the CAM was well established, and the plaster and a section of the shell were removed to allow the implantation of a silicone ring. The ring was placed at the upper pole, and 2 × 10^6^ cells (in 15 µL Matrigel) per egg were filled in that form to shape solid tumors. On day 12 and 14, these tumors were exposed to cold physical plasma or argon gas for 60 s or were left untreated. As a positive control for cell detachment, tumors grown on eggs were incubated with accutase or 0.1% trypsin in PBS (PAN Biotech) for 5 min. To address the question of whether plasma treatment physically detaches tumor cells during treatment that could potentially spur metastasis, tumors were rinsed with 150 µL growth medium for both treatment days. An additional plastic ring placed around the tumor prevented leakage of the solution, and hence the solution could be recovered after rinsing, and analyzed in downstream assays (on day 12 and day 14), such as absolute cell counting with flow cytometry or metabolic activity of proliferated cells 6 days after collection. Moreover, the toxic effects of plasma treatment were addressed by the surgical removal of tumors on day 14 and weight analysis using a precision balance (Sartorius).

### 2.8. Statistical Analysis

All experiments were repeated several times in independent runs (as detailed in figure legends), and data are displayed as mean with standard deviation (SD) or standard error (SEM), if not indicated otherwise. For multiple comparisons of effects between different groups, analysis of variance (ANOVA II) with Dunnet’s post-testing was utilized. Differences between two groups were detected using unpaired t-test with *Welch’s* correction to pay attention to unequal SDs. If requirements for parametric testing were not fulfilled, the *Mann-Whitney* U-test was utilized. Calculations and graphing were performed using *Prism 8.1* (GraphPad Software, La Jolla, CA, USA). Levels of significance are shown in the graphs as numerical value or asterisks (*, **, or *** for the *p*-values < 0.05, < 0.01, or < 0.001, respectively).

## 3. Results

### 3.1. Plasma Treatment Reduced Cellular Metabolism, Growth, and Motility in vitro

**Figure 1:**
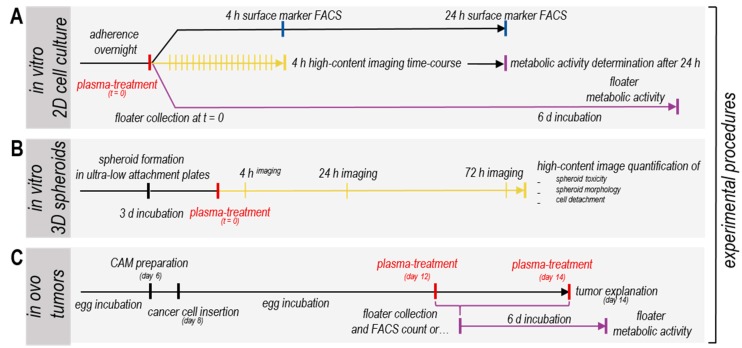
Schematic overview of the experimental setup. Experimental procedures for analysis of plasma effects on four human pancreatic cancer cell lines (**A**) in vitro using 2D cultures, (**B**) in vitro using 3D tumor spheroids and (**C**) in ovo using solid tumors. Time-points of experimental downstream analysis or high-content imaging are indicated as bars.

**Figure 2:**
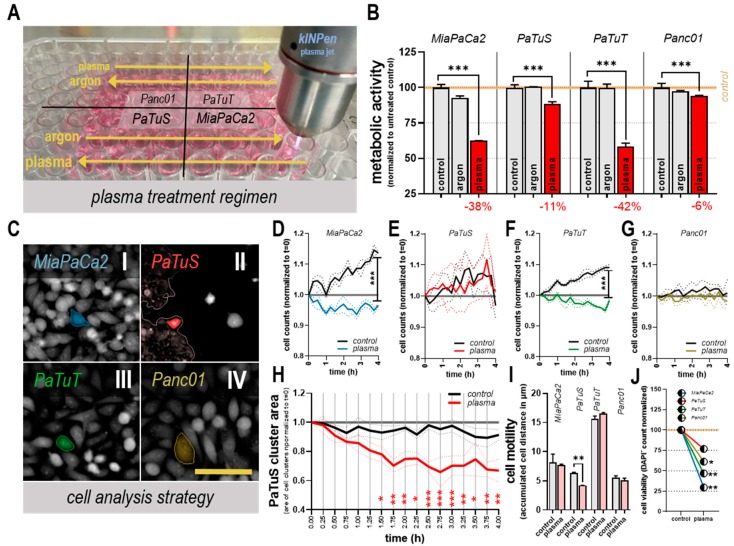
Plasma treatment reduced cellular metabolism, growth, and motility in vitro. (**A**) treatment of MiaPaCa2, PaTuS, PaTuT, and Panc01 human pancreatic cancer cells with the kINPen argon plasma jet utilizing an xyz table for standardization of plasma-treatment conditions (driving directions and conditions indicated as yellow arrows); (**B**) metabolic activity of cancer cells 24 h after argon gas or plasma exposure for 60 s (numerical value of the reduction is shown in red) normalized to each respective untreated control; (**C**) digital phase-contrast images of pancreatic cancer cells and representative cell tracking and cluster detection (light red in PaTuS cells) via quantitative image analysis; cell counts during a 4 h time course normalized t = 0 of control and plasma-treated (**D**) MiaPaCa2, (**E**) PaTuS, (**F**) PaTuT, and (**G**) Panc01 cells; (**H**) cluster area of PaTuS cell during the time-laps normalized on t = 0; (**I**) mean accumulated distance of cells 4 h post-treatment; and (**J**) DAPI^-^ cells normalized on respective untreated control 24 h after plasma exposure counted via flow cytometry. All data are mean + or ± SEM (except **J**, mean only) and are representatives of four independent experiments; scale bar represents 100 µm.

**Figure A1:**
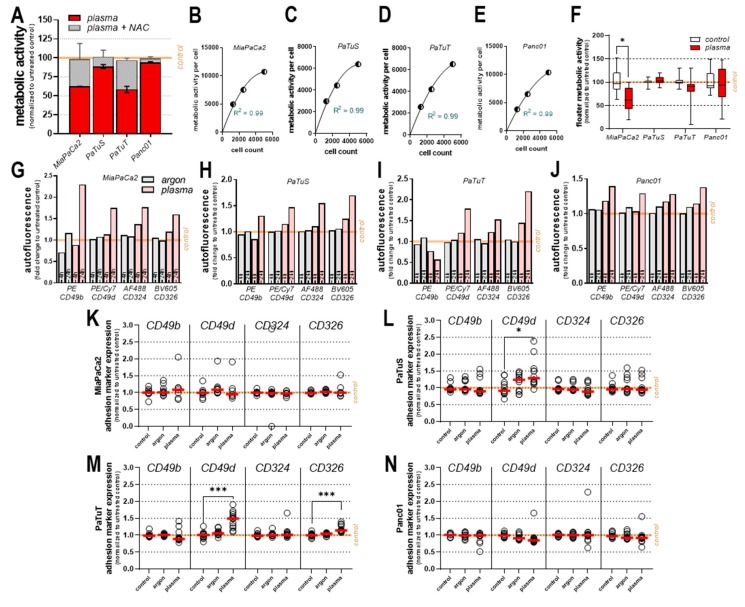
Plasma treatment affected cellular morphology and expression of early adhesion markers, and plasma’s toxicity is quenched with scavenger n-acetylcysteine. (**A**) decrease of metabolic activity 24 h after exposure to physical plasma and alleviation of toxic plasma effect upon pre-incubation with n-acetylcysteine (NAC); correlation of metabolic activity and viable cell count for (**B**) MiaPaCa2, (**C**) PaTuS, (**D**) PaTuT, and (**E**) Panc01 cells with goodness of fit (R^2^) of different polynomial functions of the 2nd degree (quadratic); (**F**) metabolic activity normalized to respective untreated controls of detached cells in supernatant following treatment (floaters) subsequently allowed for proliferation in another plate for 6 days; fold change of autofluorescence 4 h and 24 h after treatment with argon gas or plasma for 60 s in channels for PE, PE/Cy7, AF488, and BV605 for (**G**) MiaPaCa2, (**H**) PaTuS, (**I**) PaTuT, and (**J**) Panc01 cells; fold change of adhesion marker expression 4 h post-treatment normalized to untreated control and corrected for autofluorescence for (**K**) MiaPaCa2, (**L**) PaTuS, (**M**) PaTuT and (**N**) Panc01 cells. Data are mean + SEM (**A**), mean (**B**–**E**,**G**–**J**), mean and single values (**K**–**N**) or median with minimum, maximum and SEM (**F**) of representative data (**A**) or several independent replicates (**B**–**N**).

Cold physical plasma is known to generate various reactive species that can interfere with cells’ homeostasis and therefore diminish their viability. To test whether this novel treatment may promote metastasis, four different pancreatic carcinoma cell lines (MiaPaCa2, PaTuS, PaTuT, and Panc01) were exposed to plasma followed by their experimental analysis (Figure 1A–C). For comparison, these cells were exposed for 60 s to argon gas or plasma, or were left untreated (Figure 2A). Plasma significantly reduced the metabolic activity of all pancreatic cancer cell lines, whereas argon gas alone left the cells overall unaffected (Figure 2B). MiaPaCa2 and PaTuT cells were highly vulnerable to the plasma treatment, while PaTuS and Panc01 were less sensitive (Figure 2B). Regardless, plasma effects were mediated via ROS release and deposition to cell culture, as the complete abrogation of cytotoxic plasma activity with the antioxidant scavenger n-acetylcysteine (NAC, Figure A1A) suggested. Furthermore, correlations of viable cell count and metabolic activity were observed (Figure A1B–E) and therefore conclusions can be drawn towards a plasma-mediated reduced viable cell count that is the reason for the decreased whole-well metabolic activity. To analyze the growth behavior in more detail, high content image analysis of the four different cell lines’ cytosolic area (Figure 2C) enabled absolute microscopy cell counting and showed a significant reduction during a 4 h live-cell time-lapse imaging analysis post-plasma treatment in MiaPaCa2 (Figure 2D) and PaTuT cells (Figure 2F). Absolute cell counts of the rather robust PaTuS (Figure 2E) and Panc01 (Figure 2G) cells were unaffected. PaTuS cells in vitro do not grow as single cells but rather form 2D monolayers/clusters. Exposure to physical plasma significantly reduced the total cluster area of these cells beginning 1.5 h after plasma treatment (Figure 2H). Such detached cells are at risk of forming metastasis, but we found a decreased accumulated distance of cells that were tracked after plasma treatment (Figure 2I). The motility of the other cell lines was unaffected by the treatment regimen (Figure 2I). Beside these impairments in cell metabolism and growth, the toxicity of the treatment regimen was validated by the observation of reduced viable cell fraction via flow cytometry (cells negative for the dead cell dye DAPI), that was significant for MiaPaCa2, PaTuT, and Panc01, and seen in tendencies for PaTuS cells (Figure 2J).

### 3.2. Plasma Treatment Modulated the Expression of Cell Adhesion Markers

**Figure 3:**
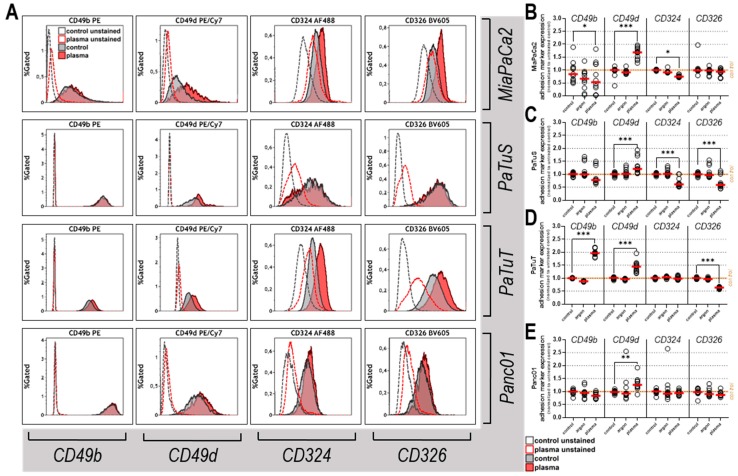
Plasma treatment modulated the expression of cell adhesion markers. (**A**) representative overlays of fluorescence intensity fluorochrome-labeled antibodies targeting CD49b, CD49d, CD324, and CD326 on human pancreatic cancer cells: unstained control cells (grey dashed line), unstained plasma-treated cells (red dashed line), stained control cells (gray histogram), and stained plasma-treated cells (red histogram) 24 h after treatment; fold change of adhesion marker expression normalized to untreated control and corrected for autofluorescence values for (**B**) MiaPaCa2, (**C**) PaTuS, (**D**) PaTuT, and (**E**) Panc01 cells. Data are mean (red line) and single values (circles) of four independent experiments.

The loss of adhesion molecules, such as integrins (CD49b, CD49d) and cadherins (CD324, CD326), is important for cancer cells to dislodge and form metastasis. To this end, the surface expression of the markers CD49b, CD49d, CD324, and CD326 was investigated 4h and 24h post plasma treatment. To analyze baseline expression levels of these molecules first, the staining was compared to unstained controls and a clear expression of all markers in the viable (DAPI^-^) cell fraction was found using five-color flow cytometry (Figure 3A). In all cases (except in the PE channel for plasma treatment of MiaPaCa2, PaTuS, and PaTuT), the autofluorescence of the unstained control cells was increased after plasma exposure (Figure A1G–J). This was taken into account in our final results by subtracting the autofluorescence values from the signal of stained cells matched to the treatment (stained untreated minus unstained untreated; stained plasma-treated minus unstained plasma-treated). As an early consequence of exposure of the cancer cells to physical plasma, an upregulation of surface-CD49d was detected in PaTuS and PaTuT cells. In PaTuT cells, also CD326 was increased 4 h after treatment, whereas all other markers where unaffected (Figure A1K–N). Analyzing the markers 24 h after plasma treatment in MiaPaCa2 cells, CD49b was found to be downregulated, but the expression of integrin CD49d was increased (Figure 3B). This marker was also higher expressed in plasma-treated PaTuS cells, while CD324 and CD326 were found to be decreased (Figure 3C). PaTuT cells upregulated CD49b and CD49d following exposure to plasma and showed a decrease in CD326 expression (Figure 3D). The robust pancreatic cancer cell line Panc01 was modulated to a higher expression of integrin CD49d after treatment with physical plasma (Figure 3E). To summarize, the major impact of plasma treatment was found in the upregulation of CD49d that was uniform through all different cell lines, but also a downregulation of CD324 and CD326 was detected in two cases (Table 1). The upregulation of integrin CD49d is positive, as it increases the loco-stability, whereas subtle but significant down-regulation of cadherins (CD324 and CD326) is not favorable and promoted further investigations in more complex 3D models. 

### 3.3. Plasma Treatment Altered Tumor Spheroid Morphology, and Induced Toxicity

**Figure 4:**
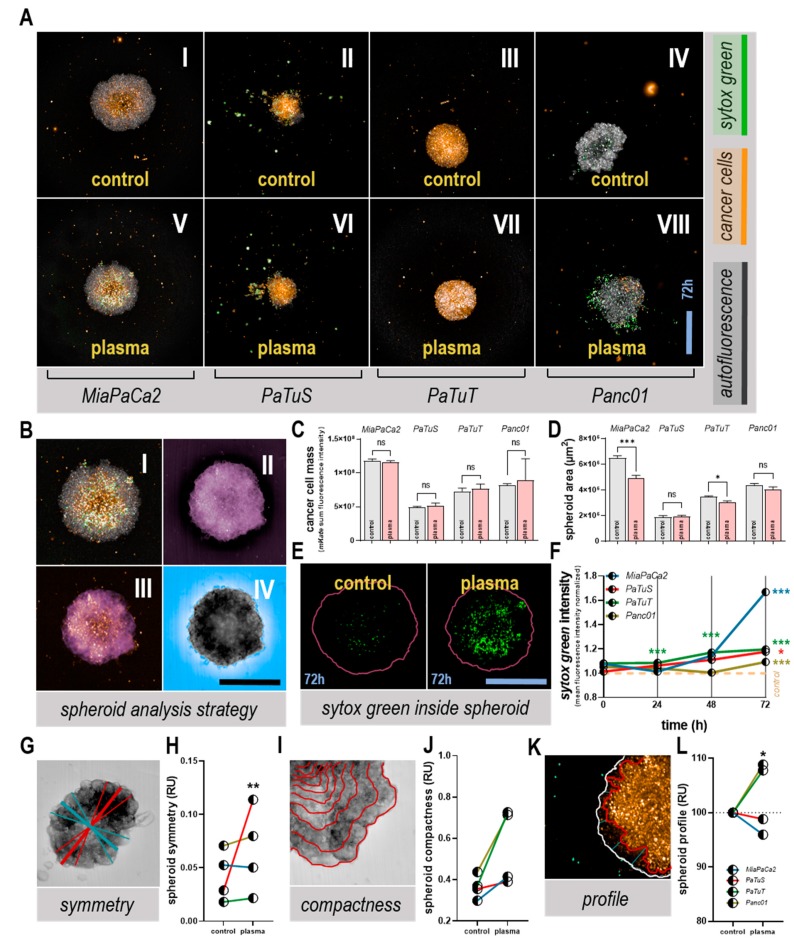
Plasma treatment altered tumor spheroid morphology and induced toxicity. (**A**) representative images of DiL-labeled tumor spheroids grown in Matrigel extracellular matrix stained with sytox green for life-dead cell discrimination 72h after exposure to plasma; (**B I**) representative merged image, (**B II**) calculated pseudo-color image of all channels, (**B III**) further processed image with intensity cut-off, and (**B IV**) spheroid-surrounding area segmented; (**C**) quantification of DiL intensity of spheroid immediately after treatment; (**D**) quantification of spheroid area segmented 72 h post plasma-treatment; (**E**) representative images of sytox green signal within the spheroid area (red line) and (**F**) their quantification during a 72 h time-course normalized on each cell line’s respective control; analysis of morphology parameters with representative scheme of (**G**) spheroid symmetry and (**H**) its quantification for all cell lines 72 h post-treatment, (**I**) spheroid compactness and (**J**) its quantification, and (**K**) spheroid profile and (**L**) its quantification. Data are mean + SEM (**C**–**D**) or mean (**F,H,J,L**) of three replicates per cell line and the group as representative of three independent experiments; ns = not significant. Scale bars represent 450 µm.

As a consequence of adhesion, marker modulation seen with plasma treatment in two-dimensional cultures remained unclear, tumor spheroids—three-dimensional structures in which cells differentiate and adhere—were utilized for further studies. Consequently, the effect of physical plasma (Figure 4A V–VIII) was compared with control spheroids (Figure 4A I–IV) up to 72 h post plasma exposure. Using high content imaging and algorithm-based quantification, stained pancreatic cancer cell spheroids were segmented, and images were merged and further processed (Figure 4B I–III) to receive information about the spheroids growing in an extracellular matrix (Matrigel) and of cells within the surrounding area of spheroids (Figure 4B IV). Immediately after plasma treatment, the number of cancer cells within the spheroid was not affected in any cell line (Figure 4C). By contrast, 72 h post-treatment, a significant area decrease in spheroids from MiaPaCa2 and PaTuT cells was observed (Figure 4D), underlining the results made with two-dimensional cultures (Figure 2B). Quantifying the fluorescence signal of the dead-cell stain sytox green within the spheroid area (Figure 4E), a time-dependent increase of the signal was detected in plasma-treated samples of all cell lines (Figure 4F). The MiaPaCa2 pancreatic cancer spheroid showed the highest toxic response following plasma treatment (Figure 4F). In addition to cytotoxic parameters, spheroids can be described by distinct morphology parameters. For example, plasma treatment significantly increased the symmetry of PaTuS spheroids (Figure 4G–H), suggesting that there is a re-organization of cells growing within the spheroid. The compactness (a relative measure of cell densities within several areas of the spheroid segmented) increased in tendency with all four human pancreatic cancer cell lines investigated, and most extremely in PaTuS and Panc01 spheroids (Figure 4I–J). This suggests a contraction of cells in response to plasma treatment, leading to higher local densities. Moreover, we identified a variation in the spheroids’ edges, and their profile was found steeper (shorter distance from the spheroid border to high cell intensities) in PaTuT and Panc01 cells following plasma treatment (Figure 4K-l). All types of analysis were algorithm-based and quantitative, taking into account thousands of individual images.

### 3.4. Plasma Treatment Unaffected or Decreased Cell Detachment from Tumor Spheroids 

**Figure 5:**
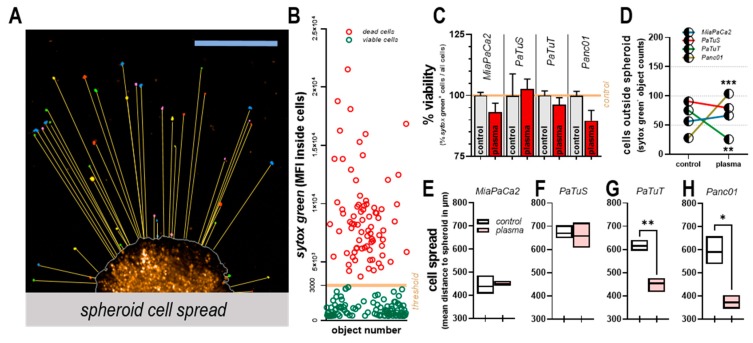
Plasma treatment unaffected or decreased cell detachment from tumor spheroids. (**A**) representative image of spheroid stained with DiL, and calculation of shortest distance from spheroid (yellow lines) to cancer cells spread in Matrigel extracellular matrix; (**B**) representative scatter dot blot of single cells detected outside the spheroid and the cut-off (orange bar) of sytox green mean fluorescence intensity (3 × 10^3^) for discrimination of viable and dead cells; (**C**) % viable cell cells (normalized to untreated controls) outside the spheroid at 72 h after plasma exposure; (**D**) total live cell counts outside the spheroid at 72 h after treatment, and mean distance of these cells (viable) to the main spheroid of (**E**) MiaPaCa2, (**F**) PaTuS, (**G**) PaTuT, and (**H**) Panc01 cells. Data are mean + SEM (**C**), mean (**D**) or mean, minimum and maximum (**E**–**H**) of three replicates per cell line and group as representative of three independent experiments; scale bar represents 450 µm.

After finding changes in integrin and cadherin expression in plasma-treated pancreatic cancer cell lines, and identifying changes in viability and morphology of three-dimensional tumor spheroids, the next step was to analyze cells detached from solid spheroids as a measure to investigate the potential promotion of metastasis with plasma treatment. This was performed in tumor spheroids being carried over 4 h after plasma treatment into a three-dimensional matrix (Matrigel) to resemble tumor cell outgrowth characteristics similar to in-vivo conditions. Hence, we term the cells within the main tumor spheroid ‘spheroid-cells’ and the cells outside the main tumor spheroid ‘matrix-cells’. A quantitative image analysis strategy was designed to detect matrix cells (Figure 5A) as well as to determine their viability via an intensity cut-off of their sytox green fluorescence signal (Figure 5B). In the latter case, we found a slight decrease in the viability of matrix-cells from MiaPaCa2, PaTuT, and Panc01 cells with plasma treatment compared to untreated controls (Figure 5C). The four different pancreatic cancer cell lines were inherently different in their capabilities of generating micro-metastases in the matrix as the number of matrix-cells varied between 25 (Panc01) and 100 (PaTuS). Three days after plasma treatment, the number of matrix cells significantly decreased compared to controls with PaTuT cells. By contrast, no changes were observed with MiaPaCa2 and PaTuS cells, whereas matrix-cells were found to be significantly increased with Panc01 cells (Figure 5D). While the total number of cells outside the spheroid is an important parameter to characterize metastatic potential, it is also important to investigate the individual cell’s distance to the main spheroid as a measure of migratory capacity through an extracellular matrix (Matrigel) from satellite metastasis. We designed an image analysis strategy to calculate individual distances for each matrix cell to address this question. In this respect, MiaPaCa2 cells (Figure 5E) showed the lowest and PaTuS (Figure 5F) the highest distance, while 60 s plasma treatment significantly and strongly decreased the mean distance of matrix cells to main spheroids in PaTuT (Figure 5G) and especially Panc01 cells (Figure 5H). Hence, despite the larger number of Panc01 matrix-cells observed with plasma treatment, these matrix cells were of low viability and had a limited migratory capacity. 

### 3.5. Plasma Treatment did not Increase Tumor Growth or Number of Viable, Detached Cells in ovo 

**Figure 6:**
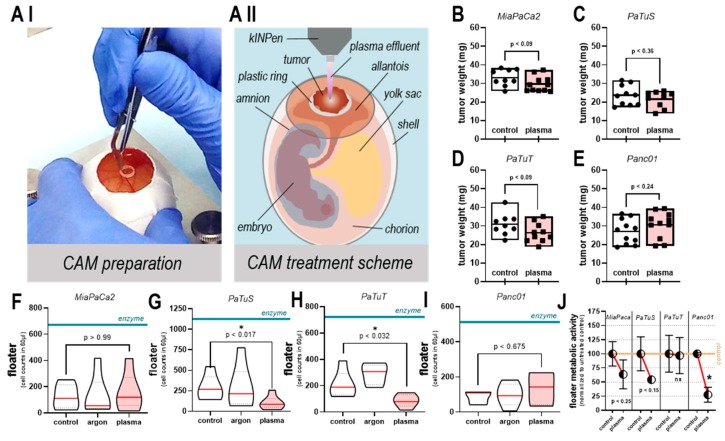
Plasma treatment did not increase tumor growth or the number of detached cells in ovo. (**A I**) preparation of a chicken egg for tumor implantation on a plastic ring; (**A II**) scheme of the anatomy of the chorion allantois membrane (CAM) and treatment of a solid pancreatic tumor tissue with the kINPen argon plasma jet (day 12 and 14); tumor weight after excision (day 14) of (**B**) MiaPaCa2, (**C**) PaTuS, (**D**) PaTuT, and (**E**) Panc01 cells; rinsing of tumors with 150 µL growth medium to collect cells detached per se or potentially through plasma treatment procedure and (**F**–**I**) the quantification of the counts of detached cells (floaters) via nuclear counterstaining and flow cytometry collected of tumors immediately after either being left untreated or treated for 60s with argon gas (argon) or plasma; enzymes (accutase or trypsin) digestion for 5 min served as a positive control (blue horizontal line); (**K**) second set of samples from (**G**–**J**) but incubated for 6 d following collection from tumors, and assessment of metabolic activity. Data are displayed as mean with the minimum, maximum, and single values (**B**–**E**) or median with minimum, maximum and quartiles as dashed lines (**F**–**I**) of at least four to five eggs per group and experiment; (**J**) are mean values ± SEM as representatives of four independent experiments.

To investigate these findings further, we used a living model system in subsequent experiments. To test the safety in terms of promotion of metastasis of plasma treatment, the physical or chemical detachment of tumor cells exposed to plasma were investigated using a realistic, innervated, macroscopic 3D-tumor mass grown on chicken embryos. (Figure 6A I). In this model, tumors were grown on the chorion allantois membrane (CAM) to analyze tumor growth and tumor spread in a complex and living system. All four human pancreatic cancer cell lines developed solid tumors that were treated repeatedly at the 12th or 14th-day post-incubation, for 60 s with plasma or argon gas, or were left untreated (Figure 6A II). On day 14, the tumors were excised, and their weight was determined. Except for Panc01, plasma treatment decreased tumor weights of all other cell lines in tendency (Figure 6B–D). Macroscopically, we did not observe metastasis (data not shown). Using the same model, a second assay was performed. Immediately after plasma treatment, the tumors were rinsed with culture medium (not draining away due to the ring placed on the egg). This was done to collect any cells physically or chemically detaching from the main tumor mass due to plasma treatment. Soon after, the medium was acquired by flow cytometry to quantify detached cells. As a positive control, solid tumors were incubated with enzymes for 5 min at 37 °C, which generated larger numbers of detached cells (floaters) in all cases (Figure 6F–I). For plasma treatment, tumors from MiaPaCa2 cells did not show a change in cell counts (Figure 6F). In contrast, in PaTuS (Figure 6G) and PaTuT cells (Figure 6H), significantly fewer cells were identified in the rinsing medium compared to untreated tumors. Low counts of floaters were detected in Panc01 tumors in all treatment groups, and no significant difference was detected between them (Figure 6I). Of note, the frequency of extra-spheroidal cells was similar in our in-ovo and 3D in-vitro model, providing good coherence and accuracy of our detection methods. As the total number of cells detached from the tumor mass does not allow conclusions on their viability and ability to proliferate, the collected medium was added to culture plates, which were allowed to incubate for 6 d before analysis of total metabolic activity per well. Detached cells from plasma-treated tumors showed a reduced metabolic activity with MiaPaCa2 (*p* < 0.25), PaTuS (*p* < 0.15), and significantly with Panc1, while PaTuT cells remained unaffected (Figure 6J). These findings are underlined by data investigating detached cells (floaters) immediately after plasma treatment in two-dimensional cultures (Figure A1F).

## 4. Discussion

Late diagnosis and metastasis are mainly responsible for high mortality in pancreatic cancer patients [31]. Although significant improvements in surgical and non-surgical treatment modalities were made, survival rates have only been modestly improving within the last 20 years from < 4% to 9% [32]. One of the reasons for poor prognosis is high rates of microscopic incomplete resections (R1-situation) [33]. The tumor side, therefore, requires a local and preferably intraoperative therapeutic option to eliminate remaining tumor cells. Vice versa, it is of prime importance for anticancer therapies not only to be effective but also safe in terms of discouraging therapy-induced tumor metastasis. Pre-clinical research suggested treatment with cold physical plasma being effective against pancreatic cancer in vitro [34,35,36,37] and in vivo [38,39,40,41]. We confirmed the cytotoxic effects of plasma treatment and addressed the question of whether plasma treatment affects pancreatic cancer cell metastasis. 

In the late 2000s, it was reported that treatment of human cells with an experimental plasma jet led to the detachment of the cells [42,43,44]. Surprisingly, the cells did not die but re-attached after some time, a potentially deleterious property for plasma cancer treatment, as this may enhance tumor metastasis. These findings prompted us to perform the current study investigating whether exposure to an accredited medical plasma jet i) generates detached tumor cells that ii) are viable and proliferate. By taking off supernatants of plasma-treated 2D monolayers and culturing them for 6 days, we confirmed that this was not the case, at least in our setting in vitro. More importantly, plasma treatment of solid macroscopic tumor mass grown on the CAM of chicken embryos failed to generate larger numbers of detached cells (compared to untreated controls) in three out of four cell lines. Even more, the long-term culture of the remaining suspended cells from plasma-treated tumors showed a decreased metabolic activity. For plasma-resistant Panc01 cells, there was a non-significant increase of detached cells with plasma treatment, which, however, was significantly impaired (>75%) concerning viability. 

The in-ovo model has several advantages. It is fast, does not need ethical approval, generates solid tumors, and vascularizes the tumors. Hence, it provides high accuracy in analyzing cell detachment. Also, matrix remodeling of solid tumors can be observed in this model [45]. The advantage of animal models is that they can provide an orthotopic environment and metastatic niche, which more resembles the in-vivo situation with tumors not being surrounded by air but matrix. However, tracing low amounts of cells metastasizing in vivo is technically challenging, and the use of human tumor cell lines requires xenograft animal models, which lack essential immune components possibly critical to metastasis. 

A critical step of tumor metastasis is the active migration of cells away from the primary tumor [46]. To investigate the influence of plasma on pancreatic tumor cell migration, we exposed 3D tumor spheroids to plasma and embedded them in a three-dimensional matrix to follow the migratory behavior of individual cells using live-cell high content imaging. Previous findings provided evidence of plasma-mediated toxicity in 3D tumor spheroids [47,48,49,50,51,52] with increased apoptosis, mitochondrial superoxide production, terminal cell death, and subsequent tumor shrinkage. The question was whether this led to metabolic reprogramming, mediated via oxidative stress, fostering a metastatic phenotype of pancreatic cancer cells that would evade the primary tumor mass into the surrounding matrix. Algorithm-based, unbiased quantification of the number and the mean distance of single and viable (sytox green-negative) cells neither showed an increased number nor increased distance with plasma treatment in three out of four cell lines. Similar to the in-ovo experiments, there was a more significant number of Panc01 cells identified in plasma-treated conditions, but these cells had a significantly reduced mean distance compared to control conditions, arguing for a resting/dormant phenotype linked to increased integrin expression [53]. These data were supported by algorithm-based tracking of 2D migration of viable plasma-treated cells showing either no change in motility or a significant decrease (PatuS). The affected cell motility can be (among others) one reason for the decreased 3D cell evasion. This phenomenon was previously observed with other cancer cell lines as well [54,55,56,57,58]. In general, cell motility is strongly linked to the expression of adhesion molecules like integrins and cadherins [59].

Moreover, integrins and cadherins are essential molecules in tumor metastasis [60]. E-cadherin (CD324) mediates cell–cell interaction, and we observed a partial but not full decrease of CD324 expression with MiaPaCa2 and PaTuS cells. Partial loss of CD324 is associated with dislodging of pancreatic cancer islets (but not single cells that can more easily enter into the bloodstream) [61] and can also be observed when culture conditions change [62]. However, absolute expression levels of CD324 were similar or increased in all four cell types when compared to non-plasma-treated controls. This was similar for EpCam (CD326), showing a relative decrease with PatuS and PatuT, while absolute values were similar or increased in all cell lines with plasma treatment. The role of EpCam (CD326) in tumor biology and metastasis is less clear, and reports find both anti-metastatic as well as pro-metastatic activity of CD326, depending on the microenvironment and tumor model [63]. Either way, the changes observed were significant but rather modest (see histograms in Figure 3, none of the changes were > ± two-fold) and there does not seem to be a correlation of cadherin expression with histological tumor grade, stage, or disease progression [64]. For integrins, we observed one increase and one decease of CD49b with one cell line, and a consistent upregulation of CD49d in all cell lines after plasma treatment. While in principle integrins mediate attachment as well as intracellular signaling upon contact with extracellular matrix components, changes in their expression may have different outcomes on tumor metastasis, depending on the type of integrin, tumor cell, and specific microenvironment [65]. The apparent difference of our data with the literature is that none of our cells are reported to be positive for CD49d, and MiaPaCa2 is reported to be devoid of CD49b [66]. However, this was reported more than a decade ago, and antibody clones, as well as fluorescence sensitivity of instrumentation, have dramatically improved since that time, especially with the use of avalanche photodiodes for the far-red channel as in our case. Studies on CD49d/α4-integrin in human pancreatic cancer are scarce, with one study reporting a decrease of tumor inflammation and growth upon the administration of CD49d-blocking antibodies in a murine, orthotopic model of pancreatic cancer [67].

## 5. Conclusions

Plasma treatment holds promises in the decelerating growth of tumor cells, including pancreatic cancer. Testing the safety of this novel therapeutic approach in vitro and in ovo, our preclinical data show plasma treatment failing to promote tumor metastasis. Future research using, e.g., animal models, RNA sequencing, and cancer patient tissue biopsies may aid in further deciphering the consequences of plasma treatment, taking into account the tumor microenvironment with immune and stromal factors.

## Figures and Tables

**Table 1:** Type and modulation of adhesion markers analyzed following plasma treatment. Type of four different adhesion molecules and their ligands, as well as the modulation of their expression 4 h or 24 h post-plasma treatment in four different human pancreatic cancer cell lines. An increase of the surface marker expression is indicated as “+”, unaffected markers as “=”, and a reduction of marker expression as “−“.

Description of Adhesion Markers Analyzed			Modulation of Adhesion Marker Expression Post Plasma Treatment				
CD	Other Names	Ligand	*MiaPaCa2*	*PaTuS*	*PaTuT*	*Panc01*	
**49b**	VLA-2α, α2 integrin	collagen	=	=	=	=	4 h
			**−**	=	**+**	=	24 h
**49d**	VLA-4α, α4 integrin	VCMA-1, MAdCAM-1, fibronectin, CD242	=	**+**	**+**	=	4 h
			**+**	**+**	**+**	**+**	24 h
**324**	E-cadherin, CDH1	CD103, E-cadherin, catenins, internalin	=	=	=	=	4 h
			**−**	**−**	=	=	24 h
**326**	Ep-Cam, TROP1	LAIR-1, LAIR-2, Ep-CAM	=	=	**+**	=	4 h
			=	**−**	**−**	=	24 h

## Data Availability

Data of this manuscript are available upon request.
